# A Novel Three Serum Phospholipid Panel Differentiates Normal Individuals from Those with Prostate Cancer

**DOI:** 10.1371/journal.pone.0088841

**Published:** 2014-03-06

**Authors:** Nima Patel, Robert Vogel, Kumar Chandra-Kuntal, Wayne Glasgow, Uddhav Kelavkar

**Affiliations:** 1 Department of Laboratory Oncology Research, Memorial University Medical Center (MUMC) and Mercer University School of Medicine (MUSM), Anderson Cancer Institute, Savannah, Georgia, United States of America; 2 Georgia Southern University, Statesboro, Georgia, United States of America; University of British Columbia, Canada

## Abstract

**Background:**

The results of prostate specific antigen (PSA) and digital rectal examination (DRE) screenings lead to both under and over treatment of prostate cancer (PCa). As such, there is an urgent need for the identification and evaluation of new markers for early diagnosis and disease prognosis. Studies have shown a link between PCa, lipids and lipid metabolism. Therefore, the aim of this study was to examine the concentrations and distribution of serum lipids in patients with PCa as compared with serum from controls.

**Method:**

Using Electrospray ionization mass spectrometry (ESI-MS/MS) lipid profiling, we analyzed serum phospholipids from age-matched subjects who were either newly diagnosed with PCa or healthy (normal).

**Results:**

We found that cholester (CE), dihydrosphingomyelin (DSM), phosphatidylcholine (PC), egg phosphatidylcholine (ePC) and egg phoshphatidylethanolamine (ePE) are the 5 major lipid groups that varied between normal and cancer serums. ePC 38:5, PC 40:3, and PC 42:4 represent the lipids species most prevalent in PCa as compared with normal serum. Further analysis revealed that serum ePC 38:5 ≥0.015 nmoles, PC 40.3 ≤0.001 nmoles and PC 42:4 ≤0.0001 nmoles correlated with the absence of PCa at 94% prediction. Conversely, serum ePC 38:5 ≤0.015 nmoles, PC 40:3 ≥0.001 nmoles, and PC 42:4 ≥0.0001 nmoles correlated with the presence of PCa.

**Conclusion:**

In summary, we have demonstrated that ePC 38:5, PC 40:3, and PC 42:4 may serve as early predictive serum markers for the presence of PCa.

## Introduction

Prostate cancer (PCa) is the most commonly diagnosed cancer in men and the second leading cause of cancer deaths in men in the western world [1], [2]. However, incidence rates of PCa differ throughout the world, suggesting that external factors, for example a high-fat diet, may contribute to disease development [3]. While PCa already poses a significant threat to the health of the U.S. population, the aging of the “baby boomer” generation will significantly exacerbate this problem [4]. The age specific incidence of PCa increases after age 60, and in 2 years, 80 million “baby boomers” will approach this milestone.

Screening for prostate cancer is controversial in light of the fact that the two major screening methods for PCa, the digital rectal examination (DRE) and the serum prostate-specific antigen (PSA) test, have limitations [5]. PSA, in combination with morphology-based factors such as clinical stage and biopsy Gleason sum, is used most commonly to diagnose and monitor prostate disease progression, but has limited efficacy due to less than ideal specificity and sensitivity. Several other PCa diagnostic and prognostic markers have been discovered and are currently being evaluated as potential adjuncts to existing screening techniques [6]. However, there remains an urgent need for the identification and evaluation of new markers to assist in early diagnosis and disease prognosis to guide clinicians in providing treatment appropriately.

Lipids play an important role in biological functions, including membrane composition and regulation, energy metabolism, and signal transduction [7], and so not surprisingly, they have been found to be involved in cancer [8]. In particular, lipids, such as phosphatidylcholine (PC) and fatty acids, play a key role PCa development and metastasis [9], [10]. Indeed, studies show an association between high dietary fat consumption and a greater risk for PCa [11], [12] as well as the potential of serum phospholipids levels to serve as predictors for PCa [13]. Since many studies have demonstrated that lipids play a critical role in PCa, the objective of our study was to investigate whether or not serum lipid profiling could discriminate between those with PCa and normal individuals, and subsequently the potential of these lipids to act as diagnostic markers for PCa screening.

## Materials and Methods

### Human serum samples from controls and individuals with PCa

This study was approved (expedited) by Memorial University Medical Center (MUMC) human subjects and ethics committee. ProMedDX, Massachusetts provided all serum samples (http://www.promeddx.com). Coded specimens were sent in a frozen state, and the laboratory personnel were blinded as to which of the specimens was from patients or normal individuals until after all of the clinical data and laboratory results became available. Initially, we analyzed the lipid profiles of 154 total serum samples: 77 from prostate cancer patients and 77 from normal subjects. For further statistical analysis, we divided serum samples into two groups: Samples from individuals 50–60 years in age and 61–70 years in age. As we were conducting an age-matched study, we excluded samples from those outside of the two age groups, which resulted in 76 normal (one sample data had an error) and 57 PCa samples. The study has been approved by the institutional review board. For detail medical history of PCa patient please refer to Data S1.

### Lipid extraction

Lipids from PCa and normal sera were extracted with chloroform and methanol, following the protocol established by the Kansas Lipidomics Research Center (KLRC); the method is an adaptation of the method described by Bligh and Dyer [14].

### Data processing

Data was processed using mass-spectrometer-specific software in conjunction with Excel.

### Electrospray ionization mass spectrometry (ESI-MS/MS) lipid profiling

An automated electrospray ionization-tandem mass spectrometry approach was used, and data acquisition and analysis were carried out as described previously [15], [16] with modifications. An aliquot of 3 µl of plasma was used. Precise amounts of internal standards, obtained and quantified as previously described [17], were added in the following quantities (with some small variation in amounts in different batches of internal standards): 0.60 nmol di12:0-PC, 0.60 nmol di24:1-PC, 0.60 nmol 13:0-lysoPC, 0.60 nmol 19:0-lysoPC, 0.30 nmol di12:0-PE, 0.30 nmol di23:0-PE, 0.30 nmol 14:0-lysoPE, 0.30 nmol 18:0-lysoPE, 0.30 nmol 14:0-lysoPG, 0.30 nmol 18:0-lysoPG, 0.30 nmol di14:0-PA, 0.30 nmol di20:0 (phytanoyl)-PA, 0.20 nmol di14:0-PS, 0.20 nmol di20:0(phytanoyl)-PS, 0.23 nmol 16:0-18:0-PI, 0.16 nmol di18:0-PI, 2.5 nmol C13:0 CE, and 2.5 nmol C23:0 CE. The sample and internal standard mixture was combined with solvents, such that the ratio of chloroform/methanol/300 mM ammonium acetate in water was 300/665/35, and the final volume was 1.2 ml. This mixture was centrifuged for 15 min at low speed to pellet particulates before presenting to the autosampler.

Unfractionated lipid extracts were introduced by continuous infusion into the ESI source on a triple quadrupole MS (API 4000, Applied Biosystems, Foster City, CA). Samples were introduced using an autosampler (LC Mini PAL, CTC Analytics AG, Zwingen, Switzerland) fitted with the required injection loop for the acquisition time and presented to the ESI needle at 30 µl/min.

Sequential precursor and neutral loss scans of the extracts produce a series of spectra with each spectrum revealing a set of lipid species containing a common head group fragment. Lipid species were detected with the following scans: PC, SM, and lysoPC, [M+H]^+^ ions in positive ion mode with Precursor of 184.1 (Pre 184.1); PE and lysoPE, [M+H]^+^ ions in positive ion mode with Neutral Loss of 141.0 (NL 141.0); PI, [M+NH4]^+^ in positive ion mode with NL 277.0; PS, [M+NH4]^+^ in positive ion mode with NL 185.0; PA, [M+NH4]^+^ in positive ion mode with NL 115.0; CE, [M+NH4]^+^ in positive ion mode with Pre 369.3. SM was determined from the same mass spectrum as PC (Pre 184.1 in positive mode) [18], [19] and by comparison with PC internal standards using a molar response factor for SM (in comparison with PC) determined experimentally to be 0.39.The collision gas pressure was set at 2 (arbitrary units). The collision energies, with nitrogen in the collision cell, were +28 V for PE, +40 V for PC (and SM), +25 V for PI, PS and PA, and +30 V for CE. Declustering potentials were +100 V for all lipids except CE, for which the declustering potential was +225 V. Entrance potentials were +15 V for PE, +14 V for PC (and SM), PI, PA, and PS, and +10 V for CE. Exit potentials were +11 V for PE, +14 V for PC (and SM), PI, PA, PS, and +10 V for CE. The mass analyzers were adjusted to a resolution of 0.7 u full width at half height. For each spectrum, 9 to 150 continuum scans were averaged in multiple channel analyzer (MCA) mode. The source temperature (heated nebulizer) was 100°C, the interface heater was on, +5.5 kV or −4.5 kV were applied to the electrospray capillary, the curtain gas was set at 20 (arbitrary units), and the two ion source gases were set at 45 (arbitrary units).

The background of each spectrum was subtracted, the data were smoothed, and peak areas integrated using a custom script and Applied Biosystems Analyst software, and the data were corrected for overlap of isotopic variants (A+2 peaks). The lipids in each class were quantified in comparison to the two internal standards of that class. The first and typically every 11^th^ set of mass spectra were acquired on the internal standard mixture only. Peaks corresponding to the target lipids in these spectra were identified and molar amounts calculated in comparison to the internal standards on the same lipid class. To correct for chemical or instrumental noise in the samples, the molar amount of each lipid metabolite detected in the “internal standards only” spectra was subtracted from the molar amount of each metabolite calculated in each set of sample spectra. The data from each “internal standards only” set of spectra was used to correct the data from the following 10 samples. Finally, the data were corrected for the fraction of the sample analyzed and normalized to the sample “dry weights” to produce data in the units nmol/mg. The result of this analysis provided a total of 354 potential lipids for early identification of the presence of PCa.

### Statistical analyses

To identify potential models using the 354 lipids that were identified, the analysis involved multiple iterations of “best subsets” logistic regression. The analysis was performed as frequently found in “high through-put” data analysis, as limiting models to no more than 3 lipids is equivalent to a genomics problem of over seven million potential biomarkers. Examples of this type of analysis are well-documented [20]–[25]. Cross-classifications and logistic regression models were employed to screen the data for potential predictor candidates. A standard approach to analysis in univariate hypothesis testing is to select an appropriate test, fix the type I error rate at a pre-specified value, decide on an appropriate level of power and determine the necessary sample size. As the analysis in this research mirrors that found in genomics, we employed the false discovery rate to help in the selection of lipids to use in the models. Statistically, the false discovery rate is the expected value of the number of type I errors divided by the number of rejected hypotheses, given at least one hypothesis is rejected [24]. The false discovery rate (FDR) is a common approach in simultaneous testing developed by Benjamini and Hochberg [26]. The FDR is commonly used in medicine and genomic studies. Once a small subset of lipids was selected, logistic regression models were constructed and compared using the lipid values as continuous variables. The final model consisted of three lipids. As the lipids were considered continuous, Receiver Operating Characteristic (ROC) curves were employed to determine optimal cut-points which allow for ease in use and interpretation [27], [28](G,H). The cut-points were determined by maximizing the area under the curve, AUC. The resultant AUC using the three lipids in the logistic regression derived composite index is 0.9157. All statistical analyses were performed using SAS 9.2™ (SAS Institute, Inc., Cary, NC.).

Please see flow Table 1 for our statistical strategy for identification of novel phospholipids.

**Table 1:** Flow chart of statistical strategy for identification of novel phospholipid.

No of age matched samples: 133 (Cases: 57, Controls: 76)
⇓
Mass Spectrometry for lipid analysis (Total No. of lipids: 354)
⇓
False discovery rate (FDR) (P-value<0.05) to control the false discoveries in multiple hypothesis testing
⇓
31 lipids were selected through FDR and used for further analysis
⇓
Odds ratio and relative risk
⇓
Final 3 lipids were selected for further analysis (ePC 38:5, PC 40:3 and PC 42:4)
⇓
Cut points decided (0.015nmole for ePC 38:5, 0.001nmole for PC40:3, 0.0001nmole for PC 42:4)
⇓
Logistic regression of Panel of three lipids (ePC 38:5, PC 40:3 and PC 42:4 for the

**Note:** Receiver Operating Characteristic Curve for accuracy of panel (Panel- 0.9157; ePC38:5- 0.7149; PC40:3- 0.8268; PC42:4-0.8509).

## Results

### Egg phosphatidylcholine (ePC 38:5), Phosphatidylcholine (PC 40:3 and PC 42:4) were identified as unique candidate for disease diagnosis

To identify specific serum lipids species associated with PCa, we performed MS analyses. Given the necessity of simultaneously comparing hundreds of lipids, we incorporated the false discovery rate (FDR) into our analyses [29], [30]. Tables 2 and 3 provide details of the aged-matched serum samples; including the Gleason scores and PSA levels for patients diagnosed with PCa (the full medical history can be found in Data S1). Samples highlighted in gray were from individuals outside of our age range and were therefore not included in the analyses. Data collected from the Kansas Lipidomics Research Center (KLRC) and processed using MS-specific software in conjunction with Excel revealed 354 different species of lipids (for details please refer Data S2). Using a FDR value of P<0.05, we identified 31 lipids statistically significantly associated with PCa (Table 4). These lipid species are from five major groups: cholester (CE), dihydrosphingomyelin (DSM), phosphatidylcholine (PC), egg phosphatidylcholine (ePC) and egg phoshphatidylethanolamine (ePE).

**Table 2:** Distribution of samples.

Age (Years)	Normal Control (n = 76)	Prostate Cancer Cases (n = 57)
50–60	30	24
61–70	46	33

**Table 3:** Age-matched prostate cancer subjects were identified with their PSA and Gleason scores (medical history) gives a baseline of study cases and controls.

Prostate Cancer Subjects						Normal Subjects	
ProMedDx	Age	PSA	Gleason	Matrix	Gender	ProMedDx	Age
number			Score			number	
**11505131**	**44**	**na**	**7**	Serum	M	11585237	50
11000244	50	3.55	7	Serum	M	11585245	50
11505133	50	na	6	Serum	M	11585299	51
11505138	53	na	7	Serum	M	11607890	52
11557623	56	na	7	Serum	M	11584113	53
11505129	55	na	7	Serum	M	11584148	53
11625321	51	na	7	Serum	M	11607800	53
11518554	52	na	6	Serum	M	11607813	53
11518558	53	na	6	Serum	M	11607832	53
11505132	53	na	6	Serum	M	11584185	54
11381070	55	na	9	Serum	M	11584945	54
11505134	56	na	6	Serum	M	11584976	55
11505135	56	na	6	Serum	M	11585046	55
11505136	56	na	6	Serum	M	11584245	56
11381068	57	na	7	Serum	M	11584286	56
11518535	57	6.8	9	Serum	M	11585303	56
11518538	57	1	6	Serum	M	11584288	57
11518550	57	na	7	Serum	M	11585153	57
11557622	57	4.9	7	Serum	M	11585314	57
11505139	58	na	8	Serum	M	11585319	57
11625323	58	na	7	Serum	M	11585351	57
11382587	59	0.1	7	Serum	M	11584933	58
11625325	59	na	6	Serum	M	11585739	58
11382594	60	na	7	Serum	M	11586140	58
11518559	60	na	6	Serum	M	11585132	59
11246504	65	na	7	Serum	M	11585521	59
11246505	65	<0.1	6	Serum	M	11609074	59
11246506	65	na	6	Serum	M	11584151	60
11505141	67	na	6	Serum	M	11585550	60
11505140	70	na	8	Serum	M	11608571	60
11518557	69	na	7	Serum	M	11584882	61
11381073	61	na	6	Serum	M	11585362	61
11382586	61	6	7	Serum	M	11583437	62
11518540	61	na	6	Serum	M	11584835	62
11518551	61	na	7	Serum	M	11585147	62
11246508	62	na	7	Serum	M	11585306	62
11381058	62	na	6	Serum	M	11585473	62
11518537	62	na	6	Serum	M	11585705	62
11518544	62	6.4	6	Serum	M	11585754	62
11382590	63	<0.1	7	Serum	M	11586037	62
11518542	63	na	7	Serum	M	11600540	62
11381062	64	na	6	Serum	M	11607895	62
11625315	64	na	7	Serum	M	11608013	62
11505143	65	na	6	Serum	M	11608056	62
11518546	65	na	7	Serum	M	11608390	62
11381072	66	na	6	Serum	M	11608457	62
11518552	66	na	9	Serum	M	11608786	62
11381069	67	na	9	Serum	M	11608876	62
11518543	67	na	6	Serum	M	11608993	62
11625319	67	3.8	7	Serum	M	11584232	63
11381071	68	na	6	Serum	M	11585732	63
11382595	68	0.8	9	Serum	M	11585753	63
11382596	68	<0.1	6	Serum	M	11608287	63
11518545	68	2.9	6	Serum	M	11608780	63
11246507	70	na	6	Serum	M	11608846	63
11382581	70	na	7	Serum	M	11608942	63
11518556	70	na	6	Serum	M	11585756	64
11625322	70	3.7	7	Serum	M	11585805	64
**11625310**	**71**	**9.7**	**6**	Serum	M	11585855	64
**11518553**	**73**	**na**	**8**	Serum	M	**11585876**	**64**
**10935542**	**72**	**1.9**	**6**	Serum	M	11600563	64
**11381063**	**71**	**3.6**	**6**	Serum	M	11608019	64
**11381064**	**71**	**na**	**6**	Serum	M	11608251	64
**11518536**	**71**	**na**	**6**	Serum	M	11608867	64
**11518547**	**71**	**na**	**6**	Serum	M	11609027	64
**11625324**	**71**	**na**	**7**	Serum	M	11566664	65
**11518539**	**73**	**5**	**6**	Serum	M	11584922	65
**11518548**	**73**	**na**	**7**	Serum	M	11585512	65
**11518549**	**74**	**na**	**6**	Serum	M	11585629	65
**11625314**	**74**	**na**	**7**	Serum	M	11585724	65
**11381065**	**79**	**3.1**	**6**	Serum	M	11608994	65
**11505142**	**80**	**na**	**6**	Serum	M	11608936	66
**11505137**	**83**	**na**	**7**	Serum	M	11608078	67
**11518555**	**81**	**na**	**7**	Serum	M	11586047	68
**11142413**	**82**	**4.9**	**6**	Serum	M	11586062	68
**11518541**	**84**	**na**	**7**	Serum	M	11585744	69
**11625311**	**84**	**na**	**7**	Serum	M	11586054	69

The bolded segment of the ProMedDx numbers are the subjects that did not fall in our age-match category.

**Table 4:** False Discovery Rate (FDR) (P-value<0.05) to control the false discoveries in multiple hypothesis testing.

Lipid molecular Species	Compound Formula	Nominal Mass	False Discovery Rate (FDR) (P<0.05)
C19:1CE	C_46_H_84_NO_2_	682.7	<.0001
C20:0CE	C_47_H_88_NO_2_	698.7	<.0001
C20:1CE	C_47_H_86_NO_2_	696.7	<.0001
C20:2CE	C_47_H_84_NO_2_	694.7	0.0014
DSM 16:0	C_39_H_81_N_2_O_6_P	705.6	0.0063
LPE 16:0	C_21_H_44_O_7_PN	454.3	0.0037
PC 38:0	C_46_H_92_O_8_PN	818.7	0.0050
PC 40:2	C_48_H_92_O_8_PN	842.7	<.0001
PC 40:3	C_48_H_90_O_8_PN	840.6	<.0001
PC 40:7	C_48_H_82_O_8_PN	832.6	0.0011
PC 42:10	C_50_H_80_O_8_PN	854.6	0.0004
PC 42:2	C_50_H_96_O_8_PN	870.7	<.0001
PC 42:3	C_50_H_94_O_8_PN	868.7	<.0001
PC 42:4	C_50_H_92_O_8_PN	866.7	<.0001
PC 42:5	C_50_H_90_O_8_PN	864.6	<.0001
PC 42:8	C_50_H_84_O_8_PN	858.6	<.0001
PC 42:9	C_50_H_82_O_8_PN	856.6	0.0002
ePC 36:1	C_44_H_88_O_7_PN	774.6	<.0001
ePC 36:5	C_44_H_80_O_7_PN	766.6	0.0040
ePC 38:1	C_46_H_92_O_7_PN	802.7	<.0001
ePC 38:2	C_46_H_90_O_7_PN	800.6	<.0001
ePC 38:3	C_46_H_88_O_7_PN	798.6	<.0001
ePC 38:5	C_46_H_84_O_7_PN	794.6	0.0007
ePC 38:6	C_46_H_82_O_7_PN	792.6	0.0053
ePC 40:2	C_48_H_94_O_7_PN	828.7	<.0001
ePC 40:3	C_48_H_92_O_7_PN	826.7	<.0001
ePC 40:4	C_48_H_90_O_7_PN	824.6	<.0001
ePC 40:5	C_48_H_88_O_7_PN	822.6	<.0001
ePE 34:1	C_39_H_78_O_7_PN	704.6	0.0001
ePE 36:3	C_41_H_78_O_7_PN	728.6	0.0072
ePE 38:0	C_43_H_88_O_7_PN	762.6	0.0022

We next determined that odds ratio and relative risk for the 31 lipid species identified by MS. Table 5 shows that the odds ratio (with 95% confidence interval [CI]) of the three lipids, ePC 38:5, PC 40:3 and PC 42:4 equals 10.061, 0.241 and 0.064, respectively. We next performed a sensitivity analysis based on these values (Table 6). For each of the individual lipids, we controlled for any confounding effects of the remaining two. For example, with PC 40:3, the odds ratio is 0.241, which indicates that after controlling the confounding effect of ePC 38:5 and PC 42:4, individuals whose level of PC 40:3 is greater than 0.001 nmoles are less likely to be “normal-appearing” as compared with those whose level of PC 40:3 is lower than 0.001 nmoles. In summary, the overall analyses strongly suggests that individuals with serum levels of ePC 38:5 ≥0.015 nmoles are more likely to be cancer-free or normal appearing, and individuals with serum levels of PC 42:4 ≥than 0.0001 nmoles are less likely to be normal as compared with those with PC 40:3 levels ≤0.001 nmoles.

**Table 5:** Estimates of odds ratio for the three lipid species ePC 38:5, PC 40:3 and PC 42:4, the reference group is the Control Group.

ePC 38.5	PC40:3	PC42:4	% Prediction	Prediction of Prostate Cancer
≤0.015	≤0.001	≤0.0001	61.84	Absent
≤0.015	≤0.001	≥0.0001	9.46	Present
≤0.015	≥0.001	≤0.0001	28.12	Present
≤0.015	≥0.001	≥0.0001	2.46	Present
**≥0.015**	**≤0.001**	**≤0.0001**	**94.22**	**Absent**
≥0.015	≤0.001	≥0.0001	51.25	Absent
≥0.015	≥0.001	≤0.0001	79.74	Absent
≥0.015	≥0.001	≥0.0001	20.25	Present

**Table 6:** Prediction of disease based on sensitivity analysis.

Odds Ratio Estimates		
Lipid species	Odds Ratio (Cases/Controls)	95% Confidence Interval
ePC 38:5	10.061	2.938–34.447
PC 40:3	0.241	0.060–0.976
PC 42:4	0.064	0.015–0.272

### Disease prediction and validity of diagnostic test

**Figure 1:**
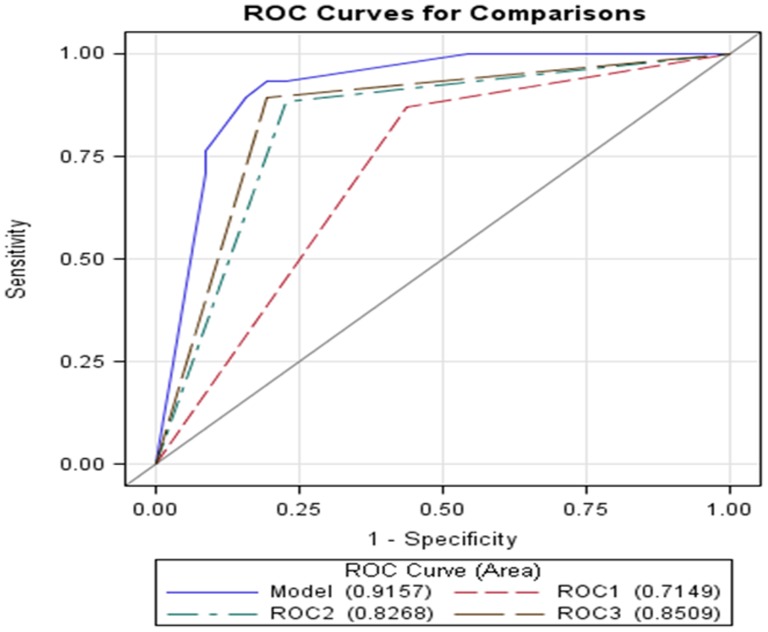
Receiver Operating Curve (ROC) for the panel of the three lipids ePC 38:5, PC40:3, and PC 42:4, for prediction of the presence or absence of PCa. X axis: 1-specificity; Y axis: sensitivity. Area under curve = 0.9157. ROC1: ---------; ROC2: -.-.-.-.; ROC3: ______ ___, and Model: _________.

We next evaluated whether ePC 38:5, PC 40:3, and PC42:4 could be used as a diagnostic test for PCa based on a sensitivity analysis (Table 7). Using logistic regression with a sensitivity of 90.20% and a specificity of 86.59%, we would predict 71 individuals as true positive, 46 as true negative, 5 as false positive, and 11 as false negative. In figure 1, we plotted a Receiver Operating Characteristic (ROC) curve to examine the true positive rate (Sensitivity) versus false positive rate (1-Specificity) [31], as a measure of the inherent validity of our diagnostic test. When we examined the three lipids individually for predicting PCa, the accuracy of using ePC 38:5 alone was 0.7149 (ROC1), for PC 40:3 was 0.8268 (ROC2), and for PC 42:4 was 0.8509 (ROC3). Looking at combinations of lipids, the ROC for PC40:3 and PC42:4 was 0.8822, for ePC 38:5 and PC42:4 was 0.9093 and for ePC 38:5 and PC40:3 was 0.8852 (data not shown). However, interestingly, using a combination of the three phospholipids (ePC 38:5, PC 40:3 and PC 42:4), resulted in an area of the curve (AUC) of 0.9157. Thus, the three lipids can be used for discriminating cancer versus normal status with an accuracy of ∼92% based on cut-off values (for their presence or absence) of 0.015 nmole for ePC 38:5, 0.001 nmole for PC 40:3, and 0.0001 nmole for PC 42:4 [8]. We thus conclude that if ePC 38:5 is present in serum sample ≥0.015 nmole and if PC 40.3 ≤0.001 nmole and PC 42:4 ≤0.0001 nmole; then we predict (95% confidence) that PCa is absent and the individual is normal. Conversely, if ePC 38:5 ≤0.015 and both PC 40:3 and PC 42:4 are greater than 0.001 and 0.0001 respectively; then the presence of PCa is very likely.

**Table 7:** Sensitivity analyses for the panel of three lipids ePC 38:5, PC 40:3 and PC 42:4 for the prediction of prostate cancer.

	Disease prediction, n = 133 (100%)	
	Normal (Positive)	Cancer (Negative)
Normal	71 (53.58%)	5 (3.76%)
n = 76	(True positive, TP)	(False positive, FP)
Cancer	11 (8.27%)	46 (34.59%)
n = 57	(False negative, FN)	(True negative, TN)
**Sensitivity** = TP/(TP+FN) = 90.20%		**Specificity** = TN/(FP+TN) = 86.59%

True positive: 71, false positive: 5, true negative: 46 and, false negative: 11; with 90.20% sensitivity and 86.59% specificity respectively.

## Discussion

Currently, the major problem in PSA testing is either over- and/or under- diagnosis. On one hand, nearly 15–25% of men have PCa even though their PSA levels are normal (4.0 ng/mL or less) [32], [33].On the other hand, high PSA levels are observed in men with benign prostate enlargement (BPH), prostatitis or indolent cancers [34], and data suggests that an estimated 40% to 50% of cases undergo unnecessary overtreatment. Unfortunately, urologists cannot embark on any specific therapeutic options unless PCa is positively identified in a biopsy, and this requires an additional 12–18 core biopsies, at a considerable cost and morbidity [35].

The report on the Prostate, Lung, Colorectal, and Ovarian (PLCO) Cancer Screening trial notes that screening was not associated with a reduction in PCa mortality during the first 7 years of the trial (rate ratio, 1.13). These results support the validity of the recent U.S. Preventive Services Task Force recommendations against screening all men over the age of 75 years [33]. Furthermore, there is no evidence that the balance of benefits and harms from PSA screening differs for African Americans and whites [36], [37]. Therefore, a major strength of this study is that the levels of ePC 38:5, PC 40:3, PC 42:4 can be used to accurately predict the presence of PCa, with a . high sensitivity of 90.20% and specificity of 86.59%. Moreover, we used age-matched samples from individuals ranging in age from 50 to 70 year; thus, this panel of lipids could differentiate between the presence and absence of PCa in individuals who were relative young. It is conceivable that if phospholipid profile is used in conjunction with PSA and DRE screening tests, there is a high likelihood of detecting PCa early-on. By using this panel as a screening test, we hope to help patients make informed decisions about whether or not to opt for surgery or other treatments that may not be necessary and that may negatively affect their quality of life.

Studies suggest that certain genetic events that can lead to malignant progression may only occur in cancer precursors (“genetic events indicative of precursor PIN”), and not in non-precursor prostatic intraepithelial neoplasia (PINs). Our previous study [38] suggests that we can distinguish the cancer precursor PINs from the benign PINs by a specific change in the 15-lipoxygenase-1 (15-LO-1) promoter DNA methylation status. Similarly, abnormalities in phospholipid metabolism can also represent hallmarks of cancer cells, especially since alterations in phospholipids are associated with malignant transformation, tumorigenicity and metastasis. Therefore, fatty acids and lipid composition can also potentially be markers of carcinogenesis [39], [40]. Previously, there has been an effort to identify candidate lipid biomarkers of PCa by shotgun lipidomics. Qualitative and quantitative profiling of six different categories of urinary phospholipids from patients with PCa were performed, but the results were inconclusive [41]. Thus, urinary metabolites may not be reliable biomarkers for PCa detection or for differentiating between indolent and aggressive tumors. Our study, however using serum shows specific differences in the phospholipid profile between individuals who lack tumors (normal) and those who have PCa.

Multiple studies have shown an association between PCa risk and diet. For example, Norrish and colleagues demonstrated that dietary fish oils may lower PCa risk, possibly through inhibition of Arachidonic acid-derived eicosanoid biosynthesis [42]. Similarly, a positive association exists between Palmitic acid and an overall risk of PCa while there is an inverse association between PCa and stearic acid [43], as well as with phosphatidylcholine [41]. Choline, an essential micronutrient necessary for cell membrane synthesis and phospholipid metabolism, also functions as an important methyl donor. Choline can modify DNA and impact cell signaling via intermediary phospholipid metabolites, influencing cell proliferation [36].

For detecting several of the fatty acids, measuring the fatty acid composition of serum phospholipids may give a better reflection of actual consumption of dietary fat than dietary assessment techniques. In fact, fatty acids in serum reflect dietary fat intake in the post-absorptive phase, so processes that affect the bioavailability of fatty acids, such as their transport, excretion, and metabolism, are taken into account [43]. Lipidomics potentially provides detailed information on a wide range of individual serum lipid metabolites. Using this approach, our study has identified potentially interesting species of cholester (CE), dihydrosphingomyelin (DSM), phosphatidylcholine (PC), egg phosphatidylcholine (ePC) and egg phoshphatidylethanolamine (ePE) that are associated with PCa. While fatty acids in adipose tissue seem to better reflect habitual dietary fat intake of some fatty acids than in blood [44], adipose tissue aspirates are more difficult to collect than blood samples in large-scale prospective studies. Moreover, adipose tissue is predominantly made up of triacylglycerol and may not be the lipid of choice for measuring fatty acids because of a smaller proportion of these fatty acids being incorporated into this lipid fraction [45].

In conclusion, because of consistency and robustness, specific phospholipids identified in our study fit the criteria for a phase 1/2 markers [46], especially if they can be combined with PSA and DRE screening for the diagnosis of PCa. Our data suggests that if the ePC 38:5 present in the serum sample is greater than 0.015 nmoles, the PC 40:3 is less than 0.001 nmoles and the PC 42:4 is less than 0.0001 nmoles, then the predictability of the absence of PCa is 94%. Conversely, if the ePC 38:5 is less than 0.015 nmoles, the PC 40:3 is greater than 0.001 nmoles, and the PC 42:4 is greater than 0.0001 nmoles, then the predictability of the presence of PCa is very high. Therefore, a combination of serum ePC 38:5, PC 40:3 and PC 42:4 can be used as a surrogate for the presence PCa. With the information gained from our study, we will continue using the lipidomics strategy in a larger data-set of normal and PCa patient serum samples to validate our findings. Limitations of this study are that the number of available samples did not allow us to divide the samples into a training sample and validation sample, there was no PSA values in the patient cohort and also no information on whether or not it was a representative patient cohort. As a result, we recognize that our model most likely overestimates the true sensitivity and true specificity. As replication is the cornerstone of all scientific research it is our hope that this work is validated with additional investigations.

## Supporting Information

Data S1 (XLSX)

Data S2 (XLSX)
